# Current and Future Potential Distribution of the Invasive Thrips *Echinothrips americanus* (Terebrantia: Thripidae) Under Global Climate Change

**DOI:** 10.1002/ece3.73636

**Published:** 2026-05-06

**Authors:** Qingling Hu, Cuiying Wang, Xianwen Yang, Fang Wang

**Affiliations:** ^1^ College of Environment and Life Sciences Weinan Normal University Weinan Shaanxi China; ^2^ Ministry of Education Key Laboratory of Molecular and Cellular Biology, Hebei Collaborative Innovation Center for Eco‐Environment, Hebei Key Laboratory of Animal Physiology, Biochemistry and Molecular Biology, College of Life Sciences Hebei Normal University Shijiazhuang Hebei China

**Keywords:** *Echinothrips americanus*, global climate change, invasive species, MaxEnt

## Abstract

*Echinothrips americanus* is an invasive pest that parasitizes over 48 families and 106 species of plants. It has spread to more than 20 countries, causing significant economic losses to the agricultural and forestry industries. Understanding the potential distribution of invasive species under climate change is crucial for management and monitoring. Therefore, this study used the Maximum Entropy (MaxEnt) Model to predict the potential distribution areas of *E. americanus* under current and future climate scenarios based on occurrence data and environmental variables. The results showed that the Annual mean temperature (Bio1) and Precipitation of the warmest quarter (Bio18) had the greatest contributions to the current distribution model of *E. americanus* . The potential distribution map revealed that the primary potential distribution areas of *E. americanus* are concentrated in Asia, Europe, North America, South America, and Africa, with a total area of approximately 3.41 × 10^7^ km^2^. Additionally, the study predicted changes in the suitable areas for *E. americanus* under future climate scenarios: the marginal suitable areas are expected to contract significantly, while the moderate and highly suitable areas may expand slightly. The total suitable area contracts more significantly under high‐emission scenarios (SSP370/SSP585) and more moderately under low/moderate‐emission scenarios (SSP126/SSP245). This study provides important data for understanding the potential global distribution of *E. americanus* and offers an early warning platform for noninfested regions that have not yet developed monitoring strategies.

## Graphical abstract

The study predicted the potential global distribution of invasive pest *Echinothrips americanus* using MaxEnt models, revealing its primary habitats concentrated in Asia, Europe, North America, South America, and Africa (total area ~ 3.41 × 10^7^ km^2^). Under future climate scenarios, marginally suitable areas are projected to contract significantly, while moderately and highly suitable areas may expand slightly, with more pronounced shrinkage under high‐emission pathways (SSP370/SSP585). These findings provide critical early warning data for noninfested regions to enhance monitoring strategies.

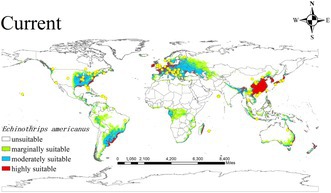

## 1 Introduction

The acceleration of global trade integration has facilitated the frequent intercontinental exchange of species with human assistance (Li et al. 2024), significantly increasing the risk of biological invasions worldwide (Shan et al. 2023). Invasive species have become one of the leading causes of global biodiversity decline, causing immense environmental and ecological disasters, substantial economic losses, severe health hazards, and food security crises, emerging as a formidable challenge for the international community, governments, and humanity (Zenni et al. 2021). It is estimated that over the past five decades, biological invasions have caused approximately US 1.288 trillion in economic losses globally, with Asia bearing the highest cost at US 430 billion. China alone accounts for about 40% of these losses, amounting to US $174.7 billion (Liu et al. 2021; Diagne et al. 2021). In particular, agricultural and forestry pests, due to the convenience of commodity trade, have spread to new regions, causing severe damage to agroforestry and resulting in economic losses amounting to billions of dollars worldwide (Liebhold et al. 2018; Wei et al. 2020; Shan et al. 2023).

Order Thysanoptera, commonly known as thrips, derives its name from the slender, fringed hairs that border their narrow wings. This order comprises approximately 6500 species from 790 genera (Mound 2024). Thrips exhibit diverse feeding habits, including phytophagous, mycophagous, and predatphagous, with over half being phytophagous (Han 1997). Many species are significant agricultural and forestry pests, capable of causing direct damage to plants and transmitting plant viruses. *Echinothrips americanus* Morgan, an invasive species belonging to the subfamily Thripinae of family Thripidae, is extremely small (adults ca. 1.2–1.5 mm) and highly cryptic, with adults and nymphs inhabiting protected microhabitats such as leaf undersides, flower buds, shoot tips, and plant crevices. This cryptic behavior severely impairs chemical control efficacy, as contact insecticides fail to reach the hidden individuals, representing a major challenge in its management (Oetting and Beshear 1993; Varga et al. 2010; Wei et al. 2010). In the early stages of infestation, it is easily overlooked. Once the opportunity for early prevention and control is missed, the pest population can multiply rapidly, resulting in chlorosis and scorching of crop leaves, reduced photosynthetic efficiency, yield losses of 10%–40%, and a 20%–40% decline in commodity value, causing significant economic losses to protected horticulture, floriculture, and fruit tree industries (Morse and Hoddle 2006; Kumar et al. 2016; Wu et al. 2022).

*E. americanus* was first reported in 1913 on *Veratrum viride* in Florida, USA (Morgan 1913). By 1968, it had spread geographically to most of the eastern United States (Stannard 1968). Although present earlier, it was first recognized as a crop pest in the 1980s, when it caused serious damage to greenhouse‐grown *Euphorbia pulcherrima* (Oetting 1987). Its distribution subsequently expanded across the U.S. and into southern Canada, both in outdoor habitats and greenhouses (Shipp and Gillespie 2001; Ferguson and Shipp 2002). It was first introduced to Europe in 1989 (Collins 1998) and rapidly invaded most European countries, including the Netherlands, France, Ireland, Italy, Poland, Austria, Sweden, Slovenia, Slovakia, Serbia, Croatia, and parts of Russia. Bulgaria intercepted the species in 2004, but no established populations have been reported domestically (Dunne and O'Connor 1997; Baranowski 1998; Marullo and Pollini 1999; Kahrer and Lethmayer 2000; Vierbergen and Cean 2006; Izhevskii 2008; Šimala and Milek 2008). In the Caribbean, it was found on soybeans in Puerto Rico (Viteri and Cabrera 2009). Its first Asian record was in 2000 near Bangkok, Thailand (Mound 2000), followed by reports from Japan (Itoh and Oguri 2003) and Java, Indonesia (Mound 2009). In China, it has been recorded in Beijing and Yangling, Shaanxi Province (Mirab‐balou and Chen 2010; Wei et al. 2010; Zhang 2010). *E. americanus* is a polyphagous insect with a wide range of host plants, including approximately 106 species from 48 families (Varga et al. 2010). It primarily damages ornamental plants, greenhouse crops, and a few field crops. Both adults and nymphs feed on plant leaves and flowers, creating white speckled spots on the leaf and floral surfaces and leaving behind black granular excrement (Trdan et al. 2008), significantly reducing the economic value of ornamental plants and greenhouse vegetables. Additionally, females use their ovipositors to lay eggs inside the leaves, further damaging the foliage (Li 2014). These records highlight its global threat. Without control, it may further spread, endangering agriculture and forestry. Predicting its potential risk areas is thus a critical tool for preventing its expansion.

Species distribution models (SDMs), based on niche theory (Guisan and Thuiller 2005), relate species occurrence data to environmental variables to characterize potential suitable habitats in probabilistic terms (Elith and Leathwick 2009; Guisan et al. 2017). Although SDMs represent testable hypotheses rather than absolute observations, they have been widely validated and applied to forecast invasion risks of alien species and support preventive management (Shan et al. 2023). Commonly employed suitability analysis software includes Climex, GARP, DIVA‐GIS, and MaxEnt, applied to study large animals, plants, or insects (Phillips et al. 2006). Among these, the Maximum Entropy (MaxEnt) model is particularly notable for its operational simplicity, lower sample size requirements, and superior predictive performance as indicated by AUC (Area Under Curve) analysis, making it increasingly popular in recent years for predicting the potential distribution of invasive species (Hernandez et al. 2006; Chen et al. 2025). Global climate change has been widely demonstrated to exert significant impacts on species distribution patterns (IPCC 2007). Global meta‐analyses have revealed consistent poleward and upward range shifts across numerous taxa in response to recent climate warming (Parmesan and Yohe 2003; Chen et al. 2011). Researching, modeling, and predicting the potential distribution of species under climate change will enhance ecological management (Jia et al. 2025). For invasive insects, climate change not only directly affects their distribution and abundance but also indirectly influences their population growth rates, spread pressure, and dispersal (Lantschner et al. 2014; Wei et al. 2019; Shan et al. 2023). Increasing evidence suggests that climate change will further exacerbate the naturalization of alien species and their impacts on new ecosystems and communities. Therefore, identifying potential habitat ranges under different climate change scenarios is crucial for developing strategies to limit the introduction and spread of invasive species (Shan et al. 2023).

This study employs the MaxEnt model to investigate the global potential spread risk of *E. americanus* , with three primary objectives: (i) identifying key climatic variables influencing the pest's potential distribution; (ii) predicting its global potential distribution range under current and future climate scenarios; and (iii) providing a theoretical framework for early warning and controlling its invasion and spread.

## 2 Material and Methods

### 2.1 Occurrence Records of *E. americanus*

**FIGURE 1:**
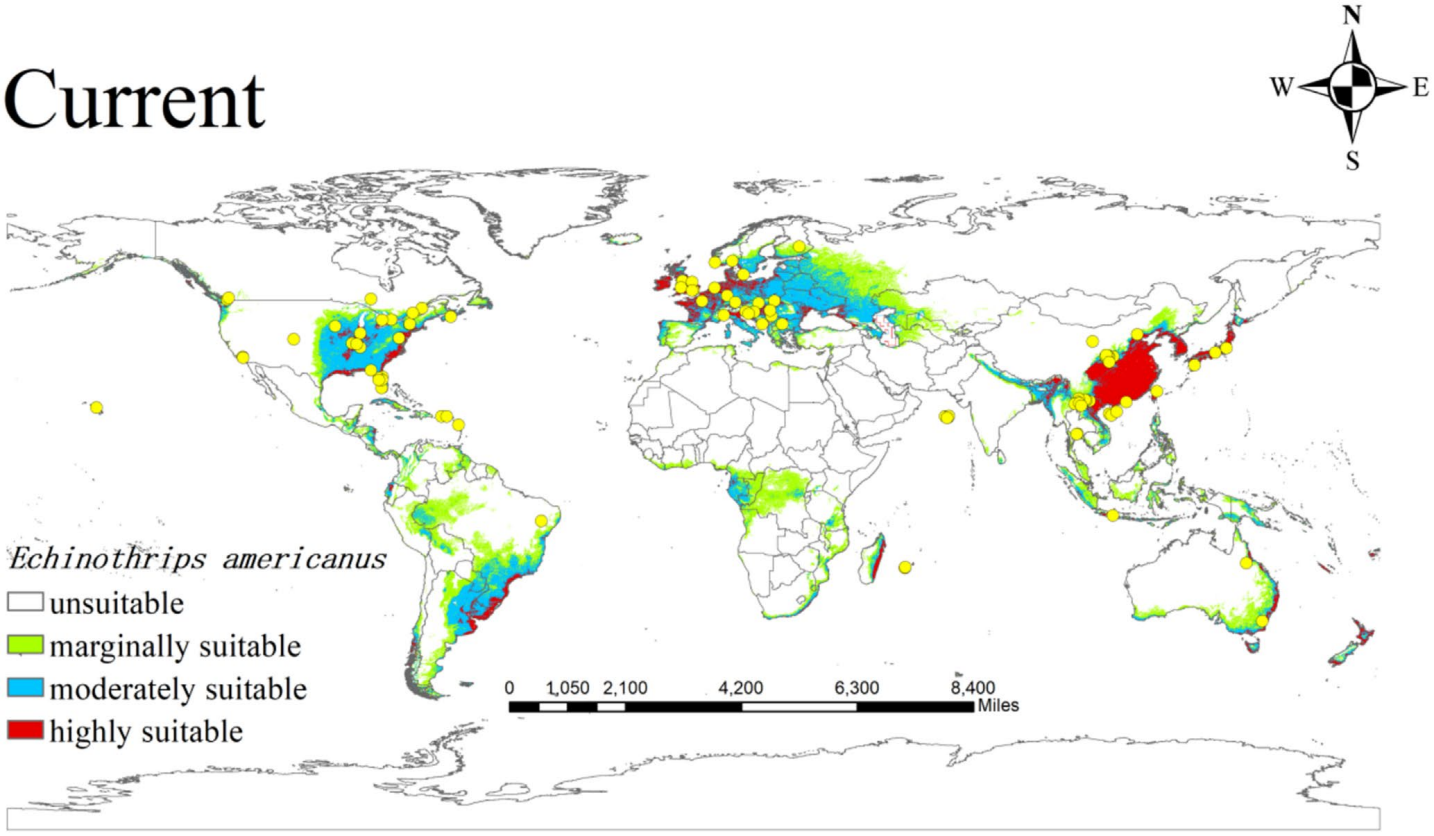
The localities and potential distribution of *Echinothrips americanus* used in the current modeling. Yellow dot, presence points used in the analyses following the filtering process. White, unsuitable; Green, marginally suitable; Blue, moderately suitable; Red, highly suitable. The base maps were created with Natural Earth Dataset (http://www.naturalearthdata.com/).

A total of 93 occurrence records of *E. americanus* were obtained from the Global Biodiversity Information Facility (GBIF, https://www.gbif.org/, Accessed 30 October 2025), ThripsWiki website (https://thrips.info/wiki/Main_Page, Accessed 25 October 2025), relevant literatures, and specimen information collected in this study (Table S1). Geographic coordinates of the distribution points were extracted using Google Earth (http://ditu.google.cn/). Considering the absence or duplication of latitude and longitude data for some species distribution points, as well as overfitting of results caused by excessively adjacent species distribution points (Qi et al. 2025), the data were filtered and verified using “spThin” in R in this study to exclude duplicate data and potential errors. Finally, 91 records were used for the construction of the distribution dataset (Table S2). The global distribution points of *E. americanus* are presented in Figure 1 using ArcGIS 10.8 (ESRI, Redlands, CA).

### 2.2 Environmental Variables

Climate change exerts profound impacts on species habitats, which may exhibit contraction, expansion, shift, or remain stable in the future (Qi et al. 2025). Species distributions at large geographic scales are primarily related to climate, and elevation may influence species distribution by affecting temperature and precipitation (Wang 2025). Climatic factors such as maximum temperature, minimum temperature, relative humidity, and precipitation were used to predict the potential global geographic distribution of a particular organism (Phillips et al. 2004). To this end, 19 bioclimatic variables (Bio1‐Bio19) from 1970 to 2000 were selected from the WorldClim database (version 2.1, http://www.worldclim.org/), along with elevation, slope, and aspect extracted from digital elevation data using QGIS v3.34.1 (https://www.qgis.org/). A spatial resolution of 2.5 arc minutes (approximately 5 km) was adopted to ensure high climatic data accuracy and optimal model performance, which were used to characterize the ecological niche of *E. americanus* in a multidimensional environmental space under current climate conditions (Table S3).

To predict the future potential global distribution of this species, we selected the BCC‐CSM2‐MR model from the latest phase (Phase 6) of the Coupled Model Intercomparison Project (CMIP6). A set of new emission scenarios integrating the characteristics of CMIP6 Shared Socioeconomic Pathways (SSPs) and considering the impacts of socioeconomic development was adopted: SSP 126 (representing a low‐emission scenario with high sustainability), SSP 245 (a moderately low‐emission scenario with a slightly slower emission reduction rate compared to SSP 126), SSP 370 (a medium‐emission scenario where insufficient measures are taken to effectively reduce greenhouse gas emissions), and SSP 585 (a high‐emission scenario characterized by the lack of effective climate policies and technological responses, leading to a significant rise in greenhouse gas emissions) (Qi et al. 2025). These emission scenarios are based on different socioeconomic assumptions and can provide more comprehensive global climate model data for the field of climate change projection. All future climate projection data (2021–2040, 2041–2060, 2061–2080, and 2081–2100) were downloaded from the WorldClim database (http://www.worldclim.org/) with a spatial resolution of 2.5 arc minutes (approximately 5 km).

In species distribution modeling, variable selection is a critical step, as environmental layers can significantly impact model performance (Shan et al. 2023). Multicollinearity among environmental variables may lead to model overfitting, which can impede the analysis of species‐environment relationships (Qi et al. 2025). Therefore, in this study, Principal Component Analysis (PCA) was performed using IBM SPSS Statistics 24 to extract six principal components (PC1–PC6), aiming to select environmental variables with low correlation but high significance levels (Table S4). Ultimately, four climatic variables (Bio 1: Annual Mean Temperature, Bio 2: Mean Diurnal Range, Bio 17: Precipitation of Driest Quarter, Bio 18: Precipitation of Warmest Quarter) and two topographic factors (elev: Elevation, aspect: Aspect) were retained to construct the species distribution model (Table 1).

**TABLE 1:** Percent contribution of each environmental variable to MaxEnt model for *Echinothrips americanus*.

Environmental variable	Percent contribution (%)
Annual mean temperature (Bio1)	62.2
Mean diurnal range (Mean of monthly (max temp − min temp)) (Bio2)	7.7
Precipitation of driest quarter (Bio17)	5.6
Precipitation of warmest quarter (Bio18)	13.9
Aspect (Asp)	1.4
Elevation (Elev)	9.3

### 2.3 Modeling Approach

**FIGURE 2:**
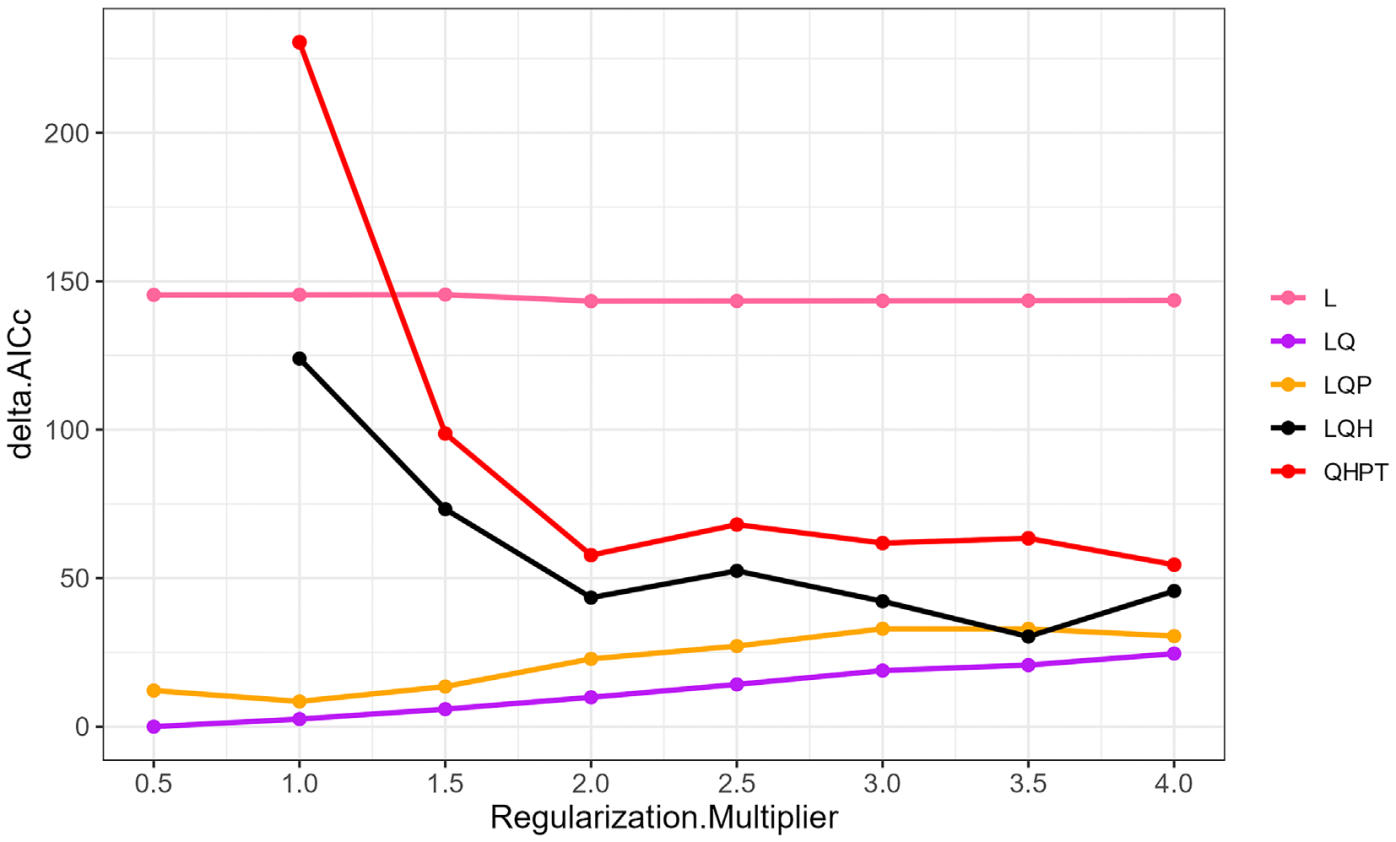
The delta Akaike information criterion coefficient (delta AICc) value of the parameter combination (FC, RM) calculated based on ENMeval.

In this study, MaxEnt software (version 3.4.1) was employed for the final analysis of the distribution of *E. americanus* . Feature classes (FC) and regularization multiplier (RM) can affect the predictive performance and accuracy of the MaxEnt model (Ji et al. 2021). The “ENMeval” package in R software (version 3.4.2, accessible via https://www.R‐project.org) was used to calculate the Akaike Information Criterion Corrected (AICc), and the parameters corresponding to the minimum AICc value from the calculation results were adopted for the MaxEnt model simulation (Qi et al. 2025). Eight different FC combinations were tested in this study, including L, LQ, LQP, QHP, LQH, LQHP, QHPT, and LQHPT (where L = linear features, Q = quadratic features, H = hinge features, P = product features, T = threshold features; auto features are an integrated setting in the model). Meanwhile, RM was set to 0.5, 1.0, 1.5, 2.0, 2.5, 3.0, 3.5, and 4.0 (Li, Liang, et al. 2023; Li et al. 2024). Ultimately, RM = 0.5 and FC = LQ (linear and quadratic features) were selected for the MaxEnt model (Figure 2, Table S5).

During the output process of MaxEnt, the convergence threshold and maximum number of iterations were set to 10^−5^ and 500, respectively. The 10‐fold cross‐validation was used to reduce select bias and avoid overfitting. More, the 10th percentile training presence threshold was used to determine the threshold separating suitable and unsuitable habitats (Wong and Yeh 2019). To interpret the reasonable simulation results of the species, the potential distribution map was reclassified into four categories: unsuitable habitat (value < threshold), low habitat suitability (threshold–0.4), moderate habitat suitability (0.4–0.6), and high habitat suitability (0.6–1.0). The final potential distribution map was generated using the Reclassification module in ArcGIS 10.8.

### 2.4 Modeling Evaluation

In this study, the performance of the MaxEnt ecological niche model was comprehensively evaluated using the Area Under Receiver Operating Characteristic Curve (AUC) and True Skill Statistic (TSS). AUC values range from 0 to 1, with higher values indicating better model predictive ability, whileTSS effectively eliminates the interference of omission error and commission error and is widely recognized as a robust evaluation index for species distribution models (Guo et al. 2019), with its value ranging from 0 to 1. A TSS value > 0.8 indicates excellent model fit, 0.6–0.8 denotes good fit, and 0.4–0.6 represents moderate fit.

## 3 Results

### 3.1 Model Performance for the Potential Distribution of *E. americanus*

The average AUC value reached 0.927, and the mean TSS value was 0.739 at the 0.05 significance level, indicating that the MaxEnt model exhibits high accuracy in predicting the current, known global geographical distribution of *E. americanus* .

### 3.2 Dominant Environmental Variables

The analysis results indicate that Bio 1 (annual mean temperature, 62.2%), Bio 18 (precipitation of the warmest quarter, 13.9%), and elevation (9.3%) are the primary variables affecting model performance. Bio 2 (mean diurnal range, 7.7%) and Bio 17 (precipitation of the driest quarter, 5.6%) are identified as less influential predictors, while aspect (1.4%) is also determined to be a predictor with minimal influence (Table 1).

### 3.3 Relationship Between the Species Distribution and Dominant Environmental Variables

**FIGURE 3:**
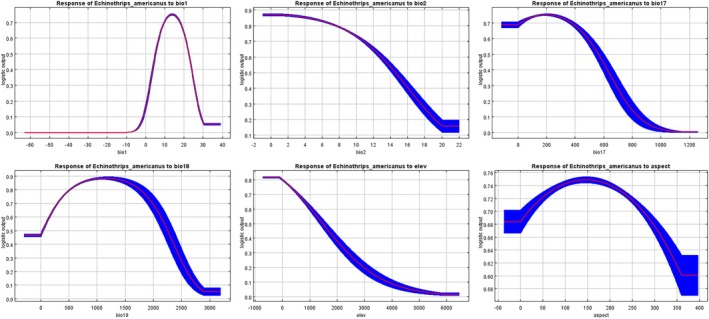
Response curves of the relationship between the species distribution and the dominant environmental variables. Bio 1, Annual mean temperature; Bio 2, Mean diurnal range (Mean of monthly (max temp − min temp)); Bio 17, Precipitation of driest quarter; Bio 18, Precipitation of warmest quarter; Elev, Elevation; Asp, Aspect.

As shown in Figure 3, the response curves in MaxEnt reveal how the climatic habitat suitability of *E. americanus* changed with respect to the six environmental factors. The environmental variables, their suitable ranges, suitability variation patterns, and key thresholds are presented in Table 2. The response curves of the distribution model indicate that the species has a higher probability of occurrence in regions with: Mean annual temperature (Bio 1): 7°C–21°C, Precipitation of the warmest quarter (Bio 18): 200–2250 mm, Elevation (elev): below 1000 m, Precipitation of the driest quarter (Bio 17): less than 500 mm, and Mean diurnal range (Bio 2): −1°C to 13°C.

**TABLE 2:** The environmental variables, their suitable ranges, suitability variation patterns, and key thresholds.

Environmental factors	Suitable ranges	Suitability variation patterns	Key thresholds
Bio 1 (annual mean temperature)	7°C–21°C	Unimodal curve, with peak suitability at 15°C	Suitability approaches zero when temperature is too low (< −10°C) or too high (> 30°C)
Bio 2 (mean diurnal range)	< 13°C	Suitability gradually decreases with increasing temperature range	Suitability drops significantly when temperature range exceeds 13°C, indicating a preference for stable temperature environments
Bio17 (precipitation of driest quarter)	< 500 mm	Suitability first increases then decreases, with moderate precipitation being more favorable	Suitability is extremely low when precipitation is > 500 mm
Bio18 (precipitation of warmest quarter)	About 1250 mm	Unimodal curve, with peak suitability at 1250 mm	Suitability declines rapidly when precipitation exceeds 2000 mm, indicating adverse effects of excessive moisture
Elevation	< 1000 m	Suitability continuously decreases with increasing elevation	Suitability is extremely low in high‐altitude areas (> 1000 m), indicating a preference for low‐altitude flat regions
Aspect	0°–350°	Insensitive to aspect changes	May be due to strong physiological mechanisms or environmental buffering capacity

### 3.4 Current Invasion Pattern of *E. americanus*

The current distribution map of *E. americanus* (Figure 1), generated based on occurrence records and six environmental variables, indicates that the suitable habitat of *E. americanus* is nearly globally distributed. Its primary potential distribution areas are concentrated in Asia, Europe, North America, South America, and Africa, with a total area of approximately 3.41 × 10^7^ km^2^.

The suitable areas are categorized into three levels based on suitability:

Highly Suitable Area: Covers an area of about ~6.42 × 10^6^ km^2^, accounting for 18.83% of the totally potential invasion area. Main distribution: Asia—eastern and southern China and adjacent regions.

Moderately Suitable Area: Covers an area of about 1.26 × 10^7^ km^2^, accounting for 36.95%. Main distribution: Asia: Other regions of China, Japan, South Korea, and parts of Southeast Asia (e.g., Vietnam, Thailand). Europe: Western Europe (e.g., UK, France), Central Europe (e.g., Germany, Poland), and Eastern Europe (e.g., European Russia). North America: Midwestern of the US and southern regions Canada.

Marginally Suitable Zone: Covers an area of about 1.51 × 10^7^ km^2^, accounting for 44.28%. Main distribution: Asia: Northwestern China, Central Asia (e.g., Kazakhstan, Uzbekistan), and West Asia (e.g., Iran, Iraq). North America: Western US (e.g., California) and northern Canada. South America: Eastern Brazil and northern Argentina. Africa: Northern Africa (e.g., Egypt, Libya) and Eastern Africa (e.g., Kenya, Tanzania).

### 3.5 Future Changes in Invasion Patterns of *E. americanus*

**FIGURE 4:**
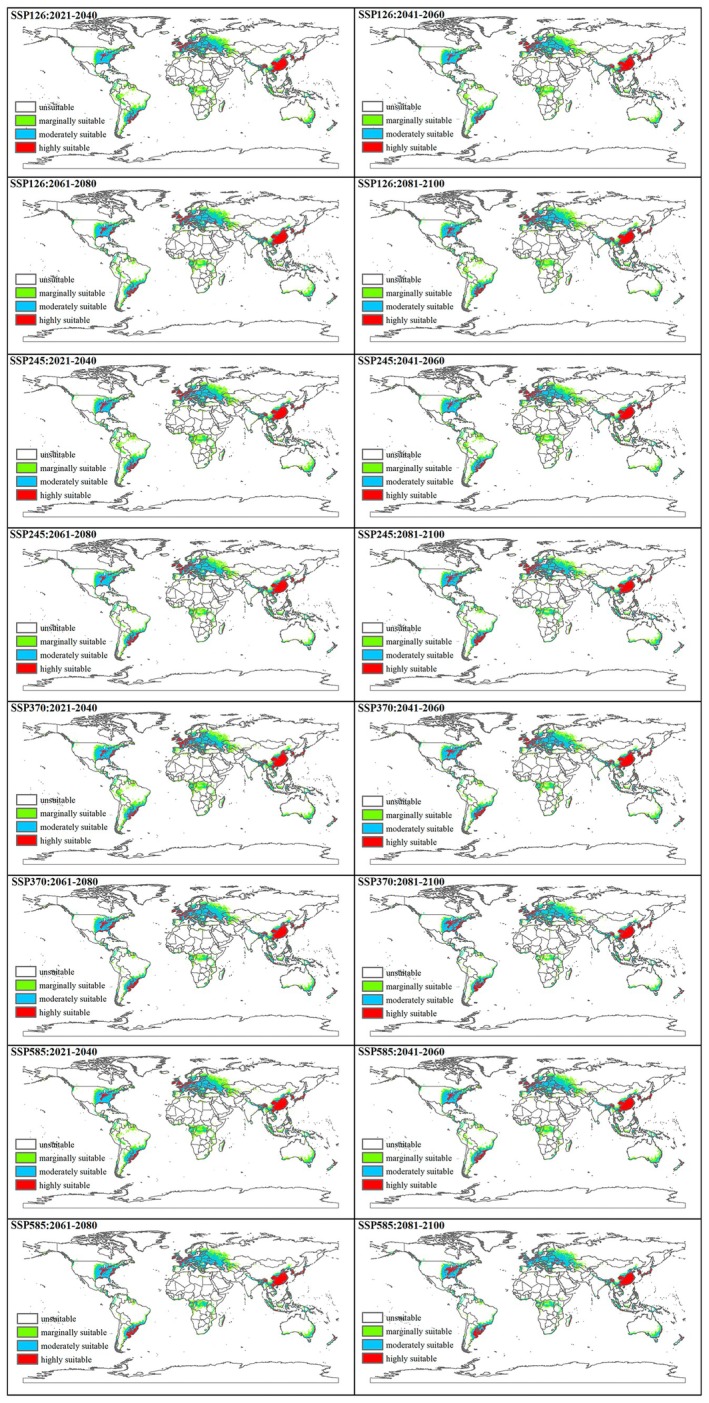
Potential distributions of *Echinothrips americanus* at the global scale under future climate scenarios (SSP 1265, SSP 245, SSP 370, and SSP 585) in 2021–2040, 2041–2060, 2061–2080, and 2081–2100. White, unsuitable; Green, marginally suitable; Blue, moderately suitable; Red, highly suitable. The base maps were created with Natural Earth Dataset (http://www.naturalearthdata.com/).

**FIGURE 5:**
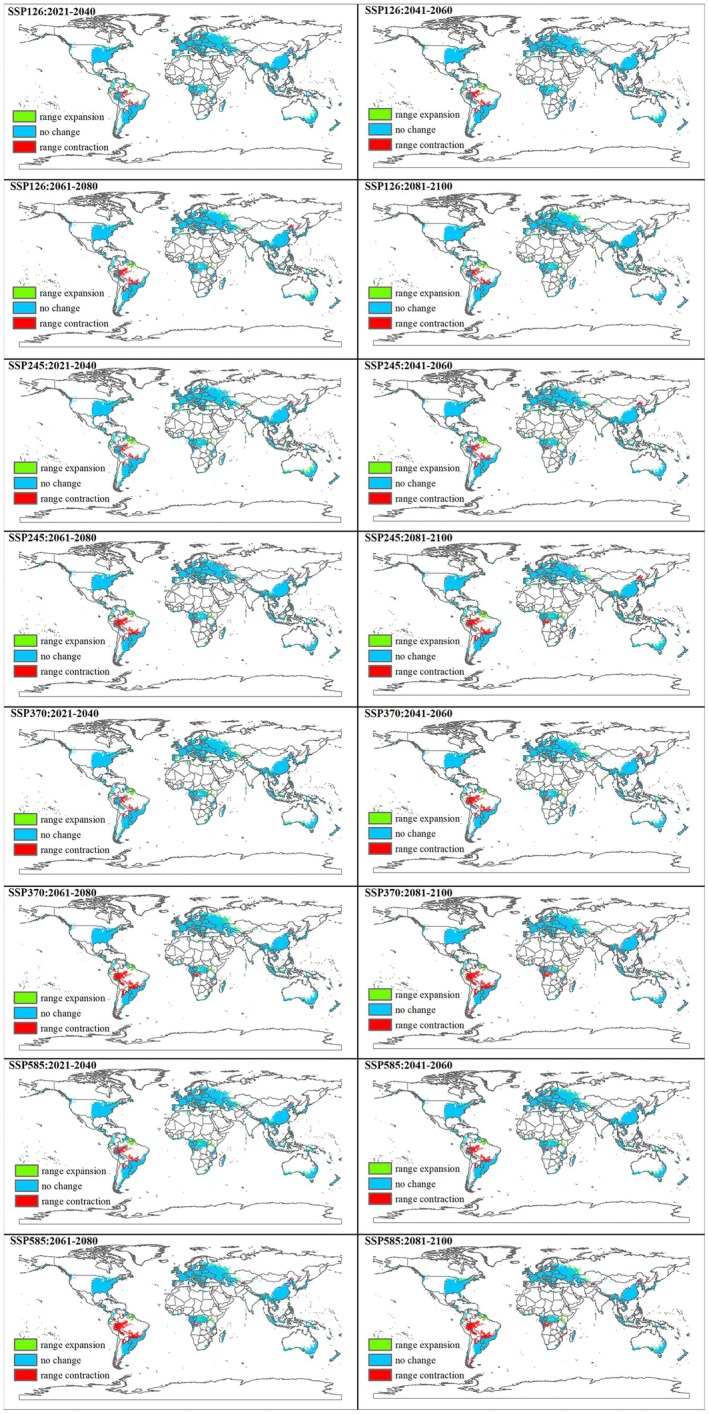
The trend of change in the total suitable distribution area of *Echinothrips americanus* under future climate scenarios (SSP 126, SSP 245, SSP 370, and SSP 585) in 2021–2040, 2041–2060, 2061–2080, and 2081–2100. (Green, range expansion; Blue, no change; Red, range contraction).

MaxEnt models for the potential distribution of *E. americanus* under future climatic scenarios (SSP 126, SSP 245, SSP 370, and SSP 585) for different periods (2021–2040, 2041–2060, 2061–2080, and 2081–2100) are shown in Figure 4 and Table 3. Figure 5 shows the changes under different climatic scenarios.

**TABLE 3:** The distribution area of *Echinothrips americanus* under current and future climate scenarios (km^2^).

Climate scenarios	Unsuitable area	Marginally suitable area	Moderately suitable area	Highly suitable area	Totally suitable area
Current	~1.14 × 10^8^	~1.51 × 10^7^	~1.26 × 10^7^	~6.42 × 10^6^	~3.41 × 10^7^
SSP126: 2021–2040	~1.15 × 10^8^	~1.32 × 10^7^	~1.29 × 10^7^	~6.72 × 10^6^	~3.29 × 10^7^
SSP126: 2041–2060	~1.16 × 10^8^	~1.24 × 10^7^	~1.28 × 10^7^	~6.81 × 10^6^	~3.20 × 10^7^
SSP126: 2061–2080	~1.16 × 10^8^	~1.22 × 10^7^	~1.30 × 10^7^	~7.06 × 10^6^	~3.22 × 10^7^
SSP126: 2081–2100	~1.15 × 10^8^	~1.30 × 10^7^	~1.31 × 10^7^	~7.08 × 10^6^	~3.31 × 10^7^
SSP245: 2021–2040	~1.14 × 10^8^	~1.39 × 10^7^	~1.30 × 10^7^	~6.97 × 10^6^	~3.39 × 10^7^
SSP245: 2041–2060	~1.15 × 10^8^	~1.33 × 10^7^	~1.29 × 10^7^	~6.85 × 10^6^	~3.30 × 10^7^
SSP245: 2061–2080	~1.17 × 10^8^	~1.13 × 10^7^	~1.28 × 10^7^	~6.77 × 10^6^	~3.09 × 10^7^
SSP245: 2081–2100	~1.17 × 10^8^	~1.04 × 10^7^	~1.37 × 10^7^	~7.36 × 10^6^	~3.14 × 10^7^
SSP370: 2021–2040	~1.15 × 10^8^	~1.35 × 10^7^	~1.28 × 10^7^	~7.02 × 10^6^	~3.34 × 10^7^
SSP370: 2041–2060	~1.16 × 10^8^	~1.12 × 10^7^	~1.34 × 10^7^	~7.13 × 10^6^	~3.17 × 10^7^
SSP370: 2061–2080	~1.17 × 10^8^	~9.77 × 10^6^	~1.38 × 10^7^	~7.30 × 10^6^	~3.09 × 10^7^
SSP370: 2081–2100	~1.19 × 10^8^	~8.07 × 10^6^	~1.34 × 10^7^	~7.38 × 10^6^	~2.88 × 10^7^
SSP585: 2021–2040	~1.15 × 10^8^	~1.34 × 10^7^	~1.29 × 10^7^	~6.73 × 10^6^	~3.30 × 10^7^
SSP585: 2041–2060	~1.16 × 10^8^	~1.12 × 10^7^	~1.34 × 10^7^	~7.04 × 10^6^	~3.16 × 10^7^
SSP585: 2061–2080	~1.18 × 10^8^	~9.53 × 10^6^	~1.35 × 10^7^	~7.02 × 10^6^	~3.00 × 10^7^
SSP585: 2081–2100	~1.19 × 10^8^	~7.78 × 10^6^	~1.41 × 10^7^	~7.33 × 10^6^	~2.92 × 10^7^

Abbreviations: SSP126, Shared Socio‐economic Pathway 126; SSP245, Shared Socio‐economic Pathway 245; SSP370, Shared Socio‐economic Pathway 370; SSP585, Shared Socio‐economic Pathway 585.

#### 3.5.1 Changes in Unsuitable Areas

The current area of unsuitable regions is approximately 1.14 × 10^8^ km^2^. Under all future SSP scenarios, unsuitable areas show an overall slow expansion trend: By 2081–2100, the area of unsuitable regions will increase to 1.19 × 10^8^ km^2^ under the SSP370 and SSP585 scenarios, representing a rise of approximately 4.39% compared to the current period.

#### 3.5.2 Changes in Suitable Areas

Suitable areas are categorized into “marginally suitable,” “moderately suitable,” and “highly suitable,” with their sum defined as the “totally suitable area.” The core trends are as follows:

##### 3.5.2.1 Marginally Suitable Areas

The current area is about 1.51 × 10^7^ km^2^. Under all future SSP scenarios, marginally suitable areas exhibit a continuous contraction: By 2081–2100, the area will decrease to 8.07 × 10^6^ km^2^ (SSP370) and 7.78 × 10^6^ km^2^ (SSP585), a reduction of approximately 46.6% and 48.5% compared to the current period, respectively.

##### 3.5.2.2 Moderately Suitable Areas

The current area is around 1.26 × 10^7^ km^2^. Under all future SSP scenarios, moderately suitable areas show a slight fluctuating increase: By 2081–2100, the area will stabilize between 1.34 × 10^7^ and 1.41 × 10^7^ km^2^ under most scenarios, representing an increase of approximately 6.3%–11.9% compared to the current period.

##### 3.5.2.3 Highly Suitable Areas

The current area is roughly 6.42 × 10^6^ km^2^. Under all future SSP scenarios, highly suitable areas display a continuous expansion: By 2081–2100, the area will increase to 7.08 × 10^6^ to 7.38 × 10^6^ km^2^ across all scenarios, a rise of approximately 10.3%–14.9% compared to the current period.

##### 3.5.2.4 Totally Suitable Areas

The current total suitable area is approximately 3.41 × 10^7^ km^2^. Future changes vary by SSP scenario: SSP126/SSP245: Show fluctuating slight contraction. By 2081–2100, the total suitable area will remain between 3.14 × 10^7^ and 3.31 × 10^7^ km^2^ (a decrease of 2.9%–9.4% compared to the current period). SSP370/SSP585: Exhibit continuous contraction. By 2081–2100, the total suitable area will drop to 2.88 × 10^7^ km^2^ (SSP370) and 2.92 × 10^7^ km^2^ (SSP585), a reduction of 15.5% and 14.4% compared to the current period, respectively.

#### 3.5.3 Core Conclusions

Under future climate scenarios, the structure of suitable areas for *E. americanus* will undergo significant transformation: marginally suitable areas will shrink sharply, while moderately and highly suitable areas will expand slightly. The total suitable area will contract more significantly under high‐emission scenarios (SSP370/SSP585), whereas the contraction will be relatively moderate under low/medium‐emission scenarios (SSP126/SSP245).

## 4 Discussion

*E. americanus* , a native species of southern USA, is a significant threat to horticultural crops, greenhouse plants, and certain economic commodities. Consequently, investigating its potential distribution is imperative. In this study, we employed the MaxEnt ecological niche model to predict the global suitable habitat of *E. americanus* , thereby clarifying the influence of climatic and elevation factors on its geographic distribution.

The model results indicate that the annual mean temperature (Bio1) ranging between 7°C and 21°C and the precipitation of the warmest quarter (Bio18) between 200 and 2250 mm are considered the most critical bioclimatic variables limiting the distribution range of *E. americanus*, with a contribution rate of 76.1%. This finding aligns with the research by Zhang (2010), which demonstrates that the growth and development of *Acanthothrips* accelerate with rising temperatures (Oetting and Beshear 1993; Zhang 2010), while the survival rates of all instars decline as temperatures exceed 22°C (Zhang 2010; Li 2014). Additionally, temperature‐induced reproductive dormancy in adult thrips has been reported (Jenser and Szenasi 2004). Elevated temperatures are known to influence insect developmental rates, survival rates, metabolic rates, and generation numbers (Logan et al. 2003). Changes in precipitation patterns under climate change may directly impact pest survival (Karuppaiah et al. 2023). In addition, small insects such as thrips are highly susceptible to heavy rainfall, which can easily wash them off host plants (Bailey 1934) and reduce their damage to plants (Elith et al. 2010). Heavy rainfall adversely affects the survival of thrips larvae (Kirk 1997) and inhibits the dispersal of adults (Lewis 1963). Nevertheless, the above impacts are temporary and localized, only affecting short‐term population dynamics, and cannot alter the overall distribution pattern of thrips determined by long‐term climatic conditions.

The prediction results from the MaxEnt model indicate that the environmental variables yield an AUC value of 0.927 and a TSS value of 0.739, demonstrating that the current model exhibits high performance, robustness, and reliability. The current prediction map reveals that the primary potential distribution areas of *E. americanus* are concentrated in Asia, Europe, North America, South America, and Africa, with a total area of approximately 3.41 × 10^7^ km^2^. The predictions of the potential distribution of *E. americanus* under four SSPs (2021–2100) show that the differences in total suitable areas across emission scenarios reflect the sensitivity of *E. americanus* to climate change: under low/moderate emission scenarios (SSP126/SSP245), the total suitable area contracts moderately, whereas under high emission scenarios (SSP370/SSP585), the contraction is significant, which aligns with the general impact of global climate change on the distribution of invasive pests. The continuous expansion of unsuitable areas under high emission scenarios may be attributed to the increased frequency of extreme climate events (e.g., heatwaves and heavy rainfall), which exceed the environmental tolerance thresholds of *E. americanus* . In contrast, the local expansion of moderately and highly suitable areas may be associated with shifts in climatic conditions in certain regions toward more favorable conditions for its survival.

In the prediction results, some areas within the assumed native distribution range of *E. americanus* were classified as “moderately suitable,” whereas some parts of its current known distribution were predicted as “unsuitable.” Such prediction biases are relatively common in species distribution models, and mainly arise from two factors. First, the model was constructed based on climatic variables and did not incorporate non‐climatic factors such as host plants, land use, human‐mediated dispersal, interspecific interactions, and microclimates. In reality, these factors strongly influence the actual distribution of thrips, which can lead to cases where the species establishes even under climatically unsuitable conditions. Second, although the climatic conditions in the native range can support the species' survival, long‐term agricultural management, natural enemy regulation, regional control measures, and other factors may limit its actual occurrence, resulting in low climatic suitability predicted by the model.

Future research should further integrate multi‐source data, such as the distribution of host plants, land use types, and natural enemy resources, to enhance the accuracy of prediction results. Simultaneously, indoor controlled experiments should be conducted to clarify the tolerance thresholds of *E. americanus* to climatic factors, including extreme temperatures and precipitation, and to reveal the physiological and biochemical mechanisms underlying its climatic adaptability. Additionally, field monitoring in high‐risk areas should be strengthened in parallel, and a population dynamics early warning system for *E. americanus* should be established to provide support for the formulation of scientific and effective prevention and control strategies.

## 5 Conclusion

In summary, we have for the first time utilized the MaxEnt model to predict the suitable habitats of *E. americanus* under both current and future scenarios, with all predictive models demonstrating high reliability and accuracy. The climatic variables Annual mean temperature (Bio1) and Precipitation of the warmest quarter (Bio18) are identified as the most critical bioclimatic factors limiting the distribution range of *E. americanus* . Under future climate scenarios, the suitable distribution of *E. americanus* exhibits a pattern of “marginal contraction and core expansion,” with the expansion of moderately and highly suitable areas potentially exacerbating its pest pressure on crops in certain regions. The significant contraction of the total suitable area under high‐emission scenarios (SSP370/SSP585) suggests that emission reduction measures can effectively mitigate its spread risk. Low/moderate emission scenarios (SSP126/SSP245) are more conducive to maintaining the stability of *E. americanus* ' distribution range, whereas high‐emission scenarios (SSP370/SSP585) may lead to a substantial contraction in its distribution range. However, the expansion of highly suitable areas in localized regions could still pose severe invasion risks. This study provides a scientific basis for identifying the invasive risk areas of *E. americanus* and formulating sustainable prevention and control strategies under the context of climate change. It also serves as a reference for predicting the potential distribution of other invasive thrips species.

## Author Contributions

**Qingling Hu:** conceptualization (equal), data curation (equal), writing – original draft (equal), writing – review and editing (equal). **Cuiying Wang:** formal analysis (equal), software (equal), writing – original draft (equal). **Xianwen Yang:** software (equal), writing – review and editing (equal). **Fang Wang:** conceptualization (equal), supervision (equal), writing – review and editing (equal).

## Funding

This study was funded by the National Natural Science Foundation of China (31900350) and the Shaanxi Provincial Department of Science and Technology Project (25JP057).

## Conflicts of Interest

The authors declare no conflicts of interest.

## Supporting information


**Table S1:** Data sources of *Echinothrips americanus* .

**Table S2:** The distribution sites of *Echinothrips americanus* .

**Table S3:** Environmental variables used for the preliminary model.

**Table S4:** Component matrix of *Echinothrips americanus* .

**Table S5:** The parameter combination results of MaxEnt model for *Echinothrips americanus* .

## Data Availability

All the required data are uploaded as [Link], [Link], [Link], [Link], [Link].
