# Supplementary material for: Current and Future Potential Distribution of the Invasive Thrips Echinothrips americanus (Terebrantia: Thripidae) Under Global Climate Change
Source: Ecol Evol. 2026 May 6;16(5):e73636. doi: 10.1002/ece3.73636 (PMC13148137; doi:10.1002/ece3.73636)
Supplement: Supplementary file 3 — Table S3: Environmental variables used for the preliminary model. [file ECE3-16-e73636-s004.docx]

Table S3. Environmental variables used for the preliminary model

| Code | Environmental variable |
| --- | --- |
| Bio 1 | Annual mean temperature |
| Bio 2 | Mean diurnal range (Mean of monthly (max temp - min temp)) |
| Bio 3 | Isothermality (Bio 2/Bio 7) (× 100) |
| Bio 4 | Temperature seasonality (standard deviation × 100) |
| Bio 5 | Max temperature of warmest month |
| Bio 6 | Min temperature of coldest month |
| Bio 7 | Temperature annual range (Bio 5–Bio 6) |
| Bio 8 | Mean temperature of wettest quarter |
| Bio 9 | Mean temperature of driest quarter |
| Bio 10 | Mean temperature of warmest quarter |
| Bio 11 | Mean temperature of coldest quarter |
| Bio 12 | Annual precipitation |
| Bio 13 | Precipitation of wettest month |
| Bio 14 | Precipitation of driest month |
| Bio 15 | Precipitation seasonality (Coefficient of Variation) |
| Bio 16 | Precipitation of wettest quarter |
| Bio 17 | Precipitation of driest quarter |
| Bio 18 | Precipitation of warmest quarter |
| Bio 19 | Precipitation of coldest quarter |
